# Injurious Effects of Tobacco, Tea, and Coffee

**Published:** 1848-10

**Authors:** 


*Injurious effects of Tobacco, Tea, and Coffee.*—Clinic of Prof. PARKER.—A man aged 35 years, has been troubled with palpitation of the heart two years; temperate habits, drinks tea and coffee, and chews tobacco; bowels slightly costive; tongue somewhat furred; pulse nearly natural; some dyspeptic uneasiness in the gastric region. The pulsations were greatly increased by mental or physical excitement, or exercise. A careful examination could detect none of the physical signs of organic disease of the heart. This case said Dr. Parker, is very interesting to the student and young practitioner, as it finely illustrates a large class of similar ailments, in which a correct diagnosis is of the greatest consequence in reference to the treatment.

There we have such a train of ordinary symptoms, embracing long continued palpitations, and these uniformly increased by mental excitement or physical exercise, that, without the aid of auscultation and percussion, it would be impossible to deny the existence of true organic disease of the heart. But if hypertrophy existed, there ought to be increased fulness of the left side; the apex of the heart ought to be found on a line outside of the nipple, or more than three inches and a half from the centre of the sternum; the hand, when placed over the heart, ought to receive the impression of being distinctly lifted up by the heart's pulsation; and there would be dulness on percussion, and absence of the respiratory murmur over a larger space than natural. Again, if the semilunar valves were diseased, application of the ear over the junction of the cartilage of the third rib with the sternum, should elicit the abnormal sounds belonging to that disease; or if the mitral valves are affected, placing the ear over the apex of the heart should elicit a similar result. There, however, we have none of these results; and hence, we may conclude with much certainty, that all the cardiac disturbance is purely functional, depending on derangement of the digestive organs,—and this again depending on the free use of *Tobacco*, *Tea*, and *Coffee*, and too much confinement within doors. What, then, are the indications of treatment? Shall we give physic in such a case? Will physic cure bad habits? Not a bit of it. Let the patient, simply, throw away his *Tobacco*, his Tea, and his Coffee; adopt a plain and wholesome diet, and take regular exercise in the open air, and he will soon be well;—in a word remove the *causes* of derangement, and the effects will cease. Dr. Parker here alluded to the fact, that much less medicine is now given by well educated physicians than formerly; and to the erroneous supposition that this was owing to the influence of some modern theories. Nothing, he said, could be further from the truth; on the contrary, it is owing entirely to the increase in our knowledge of disease, and especially to our more precise and certain means of diagnosis. For it may be laid down as a general rule, that the more certain and accurate is our knowledge of the nature, extent, and existing stage of disease, the more perfectly shall we adapt our remedies to the precise objects to be accomplished, and, consequently, the less will be required. While so long as our ideas of the nature, extent, and location of disease are confused and uncertain, so long shall we be prone to increase the quantity and variety of our remedies, with the hope that some one of the number will hit the disease. And lucky will he be, who, under such circumstances, does not hit the patient instead of the disease.—*Annalist.*

